# Innovative Strategies for Photons Management on Ultrathin Silicon Solar Cells

**DOI:** 10.1002/gch2.202300306

**Published:** 2024-02-08

**Authors:** Ning Li, Andrea Fratalocchi

**Affiliations:** ^1^ PRIMALIGHT, Faculty of Electrical and Computer Engineering, Applied Mathematics and Computational Science King Abdullah University of Science and Technology Thuwal 23955‐6900 Saudi Arabia

**Keywords:** anti‐reflection, light‐trapping, power conversion efficiency, solar cell, ultrathin silicon

## Abstract

Silicon (Si), the eighth most common element in the known universe by mass and widely applied in the industry of electronics chips and solar cells, rarely emerges as a pure element in the Earth's crust. Optimizing its manufacturing can be crucial in the global challenge of reducing the cost of renewable energy modules and implementing sustainable development goals in the future. In the industry of solar cells, this challenge is stimulating studies of ultrathin Si‐based architectures, which are rapidly attracting broad attention. Ultrathin solar cells require up to two orders of magnitude less Si than conventional solar cells, and owning to a flexible nature, they are opening applications in different industries that conventional cells do not yet serve. Despite these attractive factors, a difficulty in ultrathin Si solar cells is overcoming the weak light absorption at near‐infrared wavelengths. The primary goal in addressing this problem is scaling up cost‐effective and innovative textures for anti‐reflection and light‐trapping with shallower depth junctions, which can offer similar performances to traditional thick modules. This review provides an overview of this area of research, discussing this field both as science and engineering and highlighting present progress and future outlooks.

## Graphical abstract

Ultrathin silicon (Si) solar cell enables to decrease the tenfold thickness of conventional Si solar cells to lower the cost, and their flexibility further extends the applications in the daily life as well as the area of science and technology. However, the global challenge is enhancing the light path length during illumination to increase the ultrathin Si solar cell efficiency. This review summarizes the state of the art strategies for photon management in ultrathin Si solar cells.

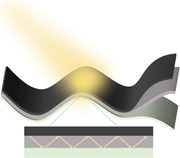

## 1 Introduction

The cost of photovoltaic (PV) power has rapidly fallen in recnt years, with an average upfront expenditure for a residential solar system between 3500 $ and 16000 $.^[^ ^1^ ^]^ Despite this figure being almost one‐third less expensive than the most economical option of fossil fuel,^[^ ^2^ ^]^ the industry of silicon (Si) solar cells still has ample opportunities for improvement in the area of material processing and manufacturing.^[^ ^3^, ^4^ ^]^ In recent years, ultrathin crystalline silicon (c‐Si) solar cells have attracted significant interest as promising candidates to further reduce costs owing to their compact size.^[^ ^5^, ^6^, ^7^ ^]^ Amorphous Si (a‐Si) is also a choice for manufacturing ultrathin Si solar cells.^[^ ^8^ ^]^ However, the higher bandgap of a‐Si than c‐Si limits its solar cell efficiency. Unlike conventional modules possessing 160 µm–180 µm thickness commonly used in PV systems, these ultrathin Si solar cells enable a tenfold decrease in the wafer thickness, reaching values below 20 µm in Si devices.^[^ ^9^ ^]^ **Table** **1** summarizes the previous works about ultrathin Si solar cells within the last 15 years. These cells offer an encouraging pathway to implement the next generation of cost‐competitive PV systems that, without subsidies, could lower the price of single modules below 0.5 $/W in the future.^[^ ^10^ ^]^ Because of their reduced thickness and increased flexibility, ultrathin cells can also extend the applications panorama of PV in aerospace, transportation, sensing, computing, and wearable electronics that conventional PV does not yet address.^[^ ^11^ ^]^

**Table 1:** Performance of ultrathin Si solar cell within 15 years.

Approach	Thickness [µm]	PCE [%]	*J*_*SC*_[*mA*~*cm* ^−2^]	*V*_*OC*_[*mV*]	FF [%]	Year	Reference
LARCs	2.8	11.22	21.98	625	81.66	2023	[24]
Inverted nanopyramid	10	15.70	33.90	589	78.50	2015	[31]
Random pyramid	16	16.40	34.0	637	75.90	2017	[16]
Inverted pyramid array	3	6.10	18.30	490	68	2016	[32]
Nanocone	sub‐10‐µm	13.70	29.0	623	76.0	2013	[5]
Nanocone textured	6.8	6.20	19.10	559	58	2013	[33]
2D periodic nanopatterning	1	4.80	15.30	435	72	2012	[36]
NPs with both ARC and BSR	8	12.40	40.10	473	−	2014	[38]
front SiNP textured with back Ag NPs	8.6	6.62	18.50	545	65.50	2014	[19]
NC‐NPs array	20	12.20	33.70	531	68.20	2016	[20]
Reconstructed‐SiNPs with BSF	20	13.63	32.10	564.10	75.20	2015	[57]
Plasmonic BR	−	7.90	15.10	810	64.50	2012	[60]
Metal holes	<1 µm	6.16	12.50	810	61	2009	[61]

While research estimates that ultrathin cells with thicknesses down to 10 µm can reach efficiencies up to 28.5%^[^ ^12^, ^13^ ^]^ and comparable to conventional c‐Si solar cells,^[^ ^9^, ^14^ ^]^ a global challenge for ultrathin cells is absorbing near‐infrared (NIR) light from a single pass of long wavelengths in the cell's absorber. Addressing this problem requires developing innovative broadband anti‐reflection coatings (ARCs) and efficient light‐trapping techniques to increase the effective optical path length inside the structure.^[^ ^15^ ^]^ Researchers explored different ARC structures manufactured with industrially scalable wet‐chemistry techniques ranging from inverted pyramid,^[^ ^16^, ^17^ ^]^ upright nanopyramid,^[^ ^18^ ^]^ nanowires,^[^ ^19^ ^]^ and dual structures,^[^ ^20^ ^]^ reaching power conversion efficiency (PCE) as high as 16.4% from solar cells with wafer thickness as low as 16 µm.^[^ ^16^ ^]^ Adding nanoparticles at the solar cell's rear side is also a strategy studied to scatter the light at an increasing optical path.^[^ ^8^, ^21^ ^]^

A central difficulty in all these methods originates from the fact that structured surfaces demand deeper junctions, accompanied by more severe carrier recombination losses.^[^ ^22^ ^]^ To address this issue, researchers recently proposed more elaborate structures surface,^[^ ^23^, ^24^ ^]^ in which a single ARC simultaneously provides increased light absorption and light scattering management. While this strategy allows reducing the cell thickness below 3 µm, the manufacturing processes of these advanced ARCs require time‐consuming and expensive e‐beam nanolithography techniques that are not rapidly scalable as wet‐chemistry.

**Figure 1:**
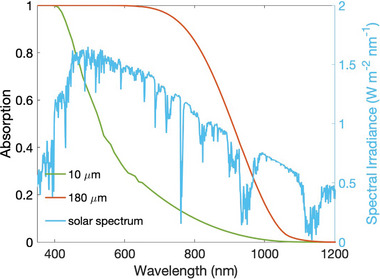
Single‐pass absorption of a planal layer of Si with varying thickness of (orange solid line) 10 µm, and (green solid line) 180 µm. We report the solar power density spectrum for reference in the solid blue line.

**Figure** **1** shows the absorption of a planar layer of Si with (orange solid line) 180 µm thickness, commercially used in solar modules, and (green solid line) 10 µm, the typical length of an ultra‐thin structure. The calculation assumes single‐pass absorption into a flat Si layer with no light path enhancement.^[^ ^25^ ^]^ The figure illustrates quantitatively the challenge of absorbing sunlight with ultra‐thin designs, which fail to capture the most intense part of the solar power density spectrum from the visible to the near‐infrared (Figure 1, blue solid line). In general, the current efficiency of 10 µm thickness thin solar cells lies around 15%, which is 5%–10% away from the current efficiency of conventionally thick Si solar cells.^[^ ^26^, ^27^, ^28^, ^29^, ^30^ ^]^ Such a performance gap offers significant research opportunities. It defines the challenge of engineering a cost‐effective structural scaling while maintaining efficient light absorption in ultrathin modules. This review summarizes recent works in this direction. It discusses ultrathin Si solar cells and the latest generation of Si‐polymer hybrid heterojunctions, analyzing optical mechanisms of light management and their manufacturing processes.

## 2 Dielectric Nanostructuring Technologies

Research groups are currently exploring diverse strategies to fabricate light management textures on ultrathin solar cells made of Si, ranging from wet chemical etching,^[^ ^31^, ^32^ ^]^ dry etching,^[^ ^33^, ^34^ ^]^ e‐beam nanolithography^[^ ^24^ ^]^ and nanoimprinting.^[^ ^35^, ^36^, ^37^, ^38^ ^]^ In the work,^[^ ^31^ ^]^ Branham et al. propose a periodic inverted pyramid texture fabricated by potassium hydroxide (KOH) wet chemistry etching technique on a 10 µm p‐type Si as a base substrate. KOH wet chemistry etching produces atomically smooth surfaces when forming surface periodic structures, minimizing the creation of unnecessary defects with detrimental recombination sites.^[^ ^39^ ^]^

**Figure 2:**
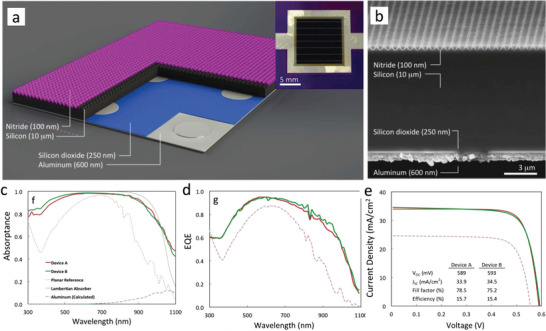
a) Optical image and schematic of a 10‐µm‐thick crystalline Si photovoltaic cell with an integrated periodic surface light‐trapping structure. b) SEM cross‐section of the complete device. c–e) Experimentally measured (c) absorptance, (d) EQE, and (e) current density–voltage (*J* − *V*) characteristics of devices A (highest efficiency) and B (highest current) and a planar reference cell. The data plotted in (c) displays total absorption, which includes parasitic absorption in the Al and Si nitride layers; simulated parasitic Al absorption plotted using results from transfer matrix method calculations. Ideal Lambertian absorption plotted for a 10‐µm‐thick Si slab using Si optical indices from ref. [74]. Reproduced with permission.^[^ ^31^ ^]^ Copyright 2015, Wiley‐VCH.

**Figure** **2a,b** shows the schematic and SEM image of the solar cell. The pyramids in ref. [31] possess a sloping angle of 54.7° and a 700 nm separation length between the centers of two adjacent elements (Figure 2b). The proposed device employs a 100 nm thick Si_3_N_4_ passivation layer for assisting light‐trapping at the surface. On the rear side, the cell uses a 250 nm SiO_2_ passivation layer for the Si, and a 600 nm thick Aluminum (Al) film working as both electrode and mirror to enhance light‐trapping further.

This work tests two implementations, A and B, of the same device structure. Figure 2c shows the light absorption of these devices. The measurements report that the inverted pyramid texturing reaches absorption larger than 90% in the wavelength range from 400 to 920 nm. In the NIR region, the device presents increased light absorption of 10% compared to earlier results.^[^ ^5^, ^40^ ^]^ The authors compare the NIR absorption to a Lambertian reference (dashed black line in Figure 2c) and observe that the inverted pyramid provides, on average, 30% higher absorption (red and green lines in Figure 2c). This work uses the transfer matrix method to simulate parasitic Al absorption, which shows that the Al back reflector (dashed blue line in Figure 2c) contributes 13% of the measured absorption at the wavelength of λ = 1100 nm. This theoretical finding implies that optimizing the back reflection could significantly enhance the device absorption on the front panel.

The authors report the external quantum efficiency (EQE) and current density‐voltage measurements to assess the impact of the texture on the final cell's performance. Figure 2d shows device A and B's EQE compared to a reference cell with no texturing. In the wavelength range from 440 to 850 nm, the EQE of A and B devices exceeds 80% (Figure 2d), which is comparable to the performances of 200 –300 µm thick Si solar cell.^[^ ^28^, ^41^ ^]^ Conversely, the EQE decreases sharply for wavelengths shorter than 450 nm (Figure 2d). The authors attribute this effect to surface Auger recombination within a heavily doped n‐type emitter that absorbs high‐energy photons. In the wavelength beyond 850 nm, the EQE of A and B drops below 80%, falling rapidly after 970 nm (Figure 2d), resulting from the parasitic photon absorption in Al and Si nitride.

Figure 2e assesses the final device performance by reporting device A and B's current density–voltage characteristics compared to a planar reference. The implementation B holds a *J*_*SC*_ of 34.5 mA cm^−2^, which is 5 mA cm^−2^ lower than the theoretical performance of of 39.6 mA cm^−2^ possessed by a 10 µm thick Lambertian absorber. The resulting efficiency of this device is 15.4%. Conversely, implementation A shows a lower current of 33.9 mA cm^−2^. However, thanks to a superior *FF* of 78.5%, device A reaches a similar efficiency of 15.7% (Figure 2e). In both devices, the *V*_*OC*_ lies in the range of ≈ 590 mV (Figure 2e). This value is lower than previously reported results,^[^ ^5^, ^22^, ^42^ ^]^ offering space for optimization in future work.

**Figure 3:**
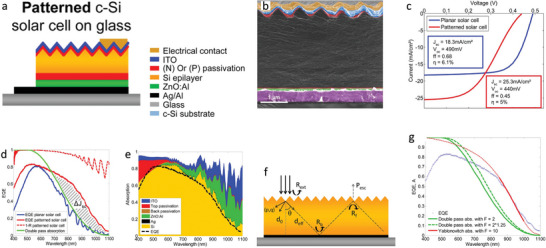
a) Complete structure of patterned c‐Si solar cell on glass. b) Colorized SEM image of the cross‐section of the complete stack of a patterned solar cell on glass. From bottom to top: glass substrate, metallic back contact/mirror (purple), ZnO:Al optical spacer (green), n‐type μc‐SiOx:H passivation layer (red), c‐Si epilayer, p‐type a‐Si:H passivation layer (red), ITO (blue), top metallic contact (yellow). c) Current–voltage characteristics of 3 µm thick planar (blue) and patterned (red) c‐Si solar cells on glass under one sun illumination. d) EQE measurements of planar and patterned solar cells, double‐pass absorption calculated for a Si slab of the same thickness d = 3 µm, and specular reflectivity R plotted as (1 – R). e) FDTD calculated absorption in each layer of a patterned solar cell (yellow: Si epilayer, black: Ag mirror, green: ZnO:Al optical spacer, brown: back passivation layer, red: top passivation layer, blue: ITO). The envelope represents the total absorption. f) Schematic of a simplified model for the solar cell. g) Comparison of the EQE of solar cells with nanopyramid arrays with different models: double‐pass absorption with *F* = 2 (flat cell) and *F* = 2 × 1.25 (effective thickness induced by diffraction), and propagation model with *F* = 10. The propagation model is plotted on the whole wavelength range (dotted red curve), with the domain of validity highlighted in solid red. Reproduced with permission.^[^ ^32^ ^]^ Copyright 2016, American Chemical Society.

Following a similar research direction, Gaucher et al. presents an inverted pyramid texture on a solar cell with 3 µm thick c‐Si epitaxial layers.^[^ ^32^ ^]^ **Figure** **3a** shows the schematic of the designed solar cell. In this work,^[^ ^32^ ^]^ the KOH wet etching yields inverted pyramids with patterned Si fabricated by a chromium hard mask and Soft UV nanoimprint lithography technique. Figure 3b illustrates the SEM image of the solar cell device. The inverted pyramid structures are arranged regularly with a depth of 330 nm. Figure 3c reports the current‐voltage characterization of the planar and patterned solar cells. At the beginning of the fabrication process in ref. [32], the starting c‐Si thickness in the solar cell is d = 3 µm. After etching, the equivalent c‐Si thickness reduces to d_eq_ = 2.75 µm. The patterend structure reports a *J*_*SC*_ of 25.3 mA cm^−2^, *V*_*OC*_ of 440 mV, *FF* = 0.45, and efficiency of 5%, while the planar control reports a *J*_*SC*_ of 18.3 mA cm^−2^, *V*_*OC*_ of 490 mV, *FF* = 0.68, and efficiency of 6.1% (Figure 3c). The texturing proposed by this work reports a 40% enhancement of *J*_*SC*_. However, its efficiency decreases more than 18% because of a lower *FF* and *V*_*OC*_.

This work studies the physical process behind the improvement of *J*_*SC*_ and the sharp decrease of other performance parameters by analyzing the spectral response of patterned solar cells and planar reference. Figure 3d shows the specular reflectivity R plotted as (1 – R) of the patterned solar cell (red dashed line), EQE measurements of planar (blue line), patterned solar cells (red line) and double‐pass absorption calculated for a Si slab of the same thickness d = 3 µm (green line). Figure 3d shows that the EQE values of the patterned solar cell are more significant than the control cell in the entire visible range. This performance increase originates from the ARC property of pyramid textures. The work compares these results with theoretical calculations from double pass absorption to further validate this claim. They evaluate the double pass absorption for a back mirror and perfect ARC by using the Beer–Lambert law:

(1) $$A\left(\lambda \right)=1-e^{-\alpha Fd}$$

where *F* is the enhancement factor of light path, *d* is the Si layer thickness, α(λ) is the wavelength‐dependent absorption coefficient for Si, and λ is the wavelength. If the pyramid array is a perfect ARC layer, the EQE values of the patterned solar cell should become lower and match the prediction from the double‐pass absorption model beyond the wavelength of 700 nm. However, the EQE obtained for the patterned solar cell outperforms the predictions from double‐pass absorption, implying that the ARC layer improves the device's EQE efficiency. The increase in the *J*_*SC*_ (Δ*J*_*SC*_) from the double‐pass absorption and the EQE of the patterned solar cell is 4.4 mA cm^−2^, representing a 63% enhancement in the *J*_*SC*_ between the planar and patterned solar cell.

To further study optical losses in the textured solar cell, the authors resort to finite‐different time‐domain (FDTD) simulation. Figure 3e shows the absorption of each layer in a patterned solar cell calculated by FDTD. The EQE (black dotted line in Figure 3e) in the patterned solar cell matches well with the theoretical prediction (yellow area in Figure 3e). By assuming ideal carriers collection from a standard AM1.5G solar spectrum, the authors estimate the solar cell to generate a 26.6 mA cm^−2^ *J*_*SC*_ from light absorption in the c‐Si layer. This value matches quite closely the measured *J*_*SC*_ of 25.3 mA cm^−2^ (Figure 3c), indicating the presence of small, additional losses from parasitic absorption and carrier recombination in passivation layers.

The authors provide additional modeling on the light‐trapping mechanism inside the solar cell to deepen the understanding of these performances. Figure 3f shows the optical model of the solar cell used in this work. Figure 3g shows the comparison of the patterned solar cells EQE with the prediction from double‐pass absorption with diverse light path enhancement factors (F): F = 2 × 1.25 from effective thickness induced by diffraction and F = 2 from a flat reference, and F = 10 from a propagation model. Theoretical results do not match the measured EQE (dashed green line in Figure 3g) in the 626 nm to 1100 nm wavelength range. This work attributes this result to the increased effective thickness resulting from the efficient light‐trapping of the cell texture. Figure 3g shows this point quantitatively, matching the EQE prediction with a varying enhancement factor from 850 to 1000 nm. The authors estimate the light path enhancement factor *F* by the normalized effective thickness *d*_*eff*_ and the possibility of photons escape *P*_*esc*_:

(2) $$F=2\frac{\bar{d_{eff}}}{d}\frac{1}{P_{esc}}$$

with the following parameters $\bar{d_{eff}}$/d = 1.25, F = 2 × 1.25 × 4 = 10, and *P*_*esc*_ is 0.25. This result predicts that the texture proposed in the work leads to an enhancement factor *F* = *F*_*r*_ · *F*_*t*_ · *F*_*l*_ from a combination of back mirror reflectivity (*F*_*c*_ = 2), ARC trapping by increased effective thickness (*F*_*t*_ = 1.25), and photon trapping in the active layer (*F*_*l*_ = 4).

**Figure 4:**
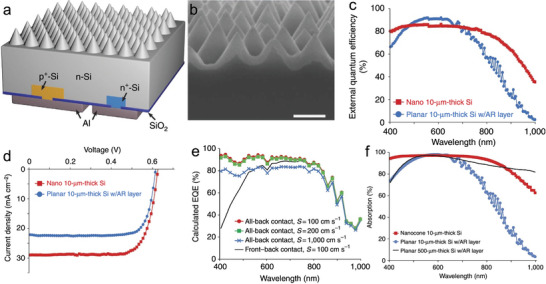
a) Schematic illustration of the solar cell device. b) SEM images of the cross‐sectional view of the nanocones. The thin layer at the top of the nanocones is an 80‐nm‐thick SiO_2_ layer. Scale bar is 400 nm. c) EQE data of the device and a planar control. d) *J* − *V* characteristics of the device and a planar control. e) Calculated EQE data of four different 10‐µm‐thick devices with the front–back‐contact and all‐back‐contact designs. f) Light absorption data of three devices. The anti‐reflection layer is Si_3_N_4_ with a thickness of 80 nm. Reproduced with permission.^[^ ^5^ ^]^. Copyright 2013, Nature Publishing Group.

In a different article,^[^ ^5^ ^]^ Jeong et al. develops a sub 10 µm all‐back‐contact ultrathin Si solar cell with nanocone textures, which achieves a PCE of 13.7%. **Figure** **4a** shows the device's schematic, with the front side patterned with a light‐trapping texture made of nanocones. Figure 4b shows the nanocones SEM image. These nanocones distribute regularly with a height of 400 nm and a diameter of 450 nm. Figure 4c shows the EQE of the solar cell (red line with square dots) against the planar control (blue line with circle dots). The EQE of the device is higher than 80% in the wavelength range from 400  to 800 nm and attains a value of 80% at the wavelength of 400 nm. As observed in previous reports, the cell absorbs short wavelengths of light near the surface, where the increased loss originates from the augmented surface area due to Auger recombination.^[^ ^43^, ^44^, ^45^ ^]^ Usually, this process results in EQEs below 50% near the wavelength of 400 nm.^[^ ^46^, ^47^ ^]^ However, contrary to this trend, the nanocone strategy developed in this work leads the solar cell to a record value EQE between 400 and 800 nm wavelengths. The main reason for this enhancement is controlling surface area. Ideally, ultrathin Si solar cell textures should minimize the surface area while improving light absorption because larger areas lead to stronger surface losses.^[^ ^48^, ^49^, ^50^ ^]^ The authors of ref. [5] calculate the surface area of nanocone structure, estimating an increase compared to a planar surface of 67%, a value that is a few orders of magnitude lower than the surface area of other reported texturing methods.^[^ ^22^, ^51^ ^]^

Figure 4d shows the *J* − *V* data of the solar cell in this work (red line with square dots) compared against a planar reference (blue line with circle dots). The designed solar cell has a *J*_*SC*_ of 29.0 mA cm^−2^, while the planar reference with 80 nm Si_3_N_4_ film as ARC layer shows a *J*_*SC*_ of 22.2 mA cm^−2^. The nanocone texture yields 30.7% enhancement of *J*_*SC*_ compared with the planar control. The authors explain this result thanks to the strong light absorption from the front side of the nanocones and the back‐contact design, which minimizes charge recombination losses. These two factors interact synergistically to provide a superior EQE performance.^[^ ^52^, ^53^ ^]^ Figure 4e shows the theoretical EQE prediction of four different 10‐µm‐thick devices obtained by computer simulations. In these calculations, when the recombination velocity (S) reaches 100 cm s^−1^, the all‐back contact device (red line with circle dots in Figure 4e) generates an EQE of 94% at 400 nm. In contrast, the front‐back contact device (black line in Figure 4e) shows a lower EQE of 28%. These calculations indicate that the all‐back‐contact design provides an additional beneficial factor that works together with the ARC properties of nanocone texturing in the range of short wavelengths.

Figure 4f reports the absorption of 10 µm thick Si with nanocone as ARC layer (red line with square dots), 10 µm thick planar control with 80 nm thick Si_3_N_4_ as ARC layer (blue line with circle dots), and 500 µm thick planar control with 80 nm thick Si_3_N_4_ as ARC layer (black line). In the wavelength range between 400 and 900 nm, the light absorption of the 10 µm thick Si with nanocones reaches 96.1%, reducing to 83.8% in the wavelength of 400 nm and 900 nm (Figure 4f). These values are 28% and 198% higher than the 10 µm thick planar control with 80 nm thick Si_3_N_4_ ARC layer, respectively. This ARC strategy shows a comparable light absorption to a 500 µm thick planar control with 80 nm thick Si_3_N_4_ ARC layer (Figure 4f). The results of this work demonstrate that surface engineering, balancing reflection, and scattering effects are important factors in enabling optimal performances.

**Figure 5:**
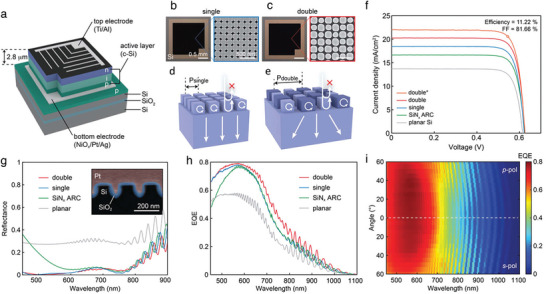
a) Schematic structure of a LARC ultrathin solar cell device. b,c) Optical microscopy image (left) and SEM image (right) of the top surface of the solar cells with (b) a single‐nanoblock anti‐reflection coating (ARC) and (c) a double‐nanoblock LARC. d,e) Schematics structure of (d) a single‐nanoblock ARC and (e) a double‐nanoblock LARC. f) *J* − *V* characteristics of solar cell devices with different photon trapping systems: a double‐nanoblock LARC (red), a single‐nanoblock ARC (blue), a conventional SiNx ARC (green), and planar reference without an ARC (grey). The orange curve shows the highest efficiency cell with a double‐nanoblock LARC on a different wafer. g) Measured reflectance spectra of ultrathin c‐Si solar cells with single (blue) and double (red) nanoblocks‐arrays, SiNx ARC (green), and without ARC (grey). Inset: SEM image of the cross‐section of the single‐nanoblock array with a thermal SiO_2_ passivation layer (light blue). h) Measured EQE spectra of ultrathin c‐Si solar cells with different light‐trapping textures. i) Measured EQE of the solar cell with the double‐nanoblock array as a function of incident angle and wavelength. Reproduced with permission.^[^ ^24^ ^]^ Copyright 2023, Wiley‐VCH.

Recently, Lee et al. propose an effective photon management strategy by light‐trapping ARCs (LARCs) for 2.8 µm thick c‐Si solar cells,^[^ ^24^ ^]^ achieving a PCE of 11.2%. **Figure** **5a** shows the schematic design of the ultrathin solar cell used. The system includes a 2.8 µm thick p–n junction‐based active layer, followed by LARCs on the device's front surface. Figure 5b,c show microscopy and SEM images of the solar cell's top surface with a single‐nanoblock ARC and a double‐nanoblock LARC, respectively. The single‐nanoblock array has nanostructures with a period of 180 nm, height of 105 nm, and width of 100 nm. The double‐nanoblock array, conversely, possesses nanostructures with a period of 450 nm, height of 105 nm, width_1_ = 100 nm, width_2_ = 145 nm. The authors use a 25 nm SiO_2_ layer in both solar cells to passivate the texture conformally.

Figure 5d,e show the schematic of the single‐nanoblock array and double‐nanoblock array, respectively. The design of the single‐nanoblock array follows Mie scattering theory by optimizing broadband ARC effects (Figure 5d). The single‐nanoblock array evolves into a double‐nanoblock array by changing the dimension of every other nanoblock in the array while closely keeping the filling fraction of Si to ≈ 30% (Figure 5e). This optimization maintains the broadband anti‐reflection property while improving light‐trapping thanks to making the unit‐cell period doubled. In Si active layer, this new design opens diffraction channels. Figure 5f reports the experimental *J* − *V* curves of the best solar cell device with different light management strategies. The resulting PCEs are 10.39% for double‐nanoblock LARC (red line), 9.41% for single‐nanoblock ARC (blue line), 8.53% for conventional SiNx ARC (green line), and 6.90 for planar reference without an ARC (grey line). The highest PCE of double‐nanoblock LARC‐based cell measured is 11.22% (orange line). The authors attribute the variance of performances in the different batches to different thicknesses of the seed Si layer.

The authors further study the enhancement of the cell performances by investigating the optical properties of double‐nanoblock LARC design. Figure 5g shows the ultrathin Si solar cells reflectance spectra with single‐nanoblock ARC (blue line) and double‐nanoblock LARC (red line), SiNx ARC (green line), and without ARC (grey line). Compared with planar control, the design with SiNx ARC shows an effective suppression near the wavelength of 600 nm with a 12.8% average reflectance. The solar cells with single‐nanoblock ARC and double‐nanoblock LARC show a better anti‐reflection than SiNx ARC at shorter wavelengths. The average reflectance is 7.1% for single‐nanoblock ARC‐based solar cells and 6.6% for double‐nanoblock LARC‐based solar cells, respectively.

Figure 5h shows the EQE curves of ultrathin solar cells with SiNx ARC (green line), without ARC (grey line), single‐nanoblock ARC (blue line) and double‐nanoblock LARC (red line). The EQE spectra variation of the solar cells with single‐nanoblock ARC, SiNx ARC, and planar reference compare well with reflectance measurements in Figure 5g. The increased EQE measured from the single‐nanoblock ARC results from its broadband anti‐reflection, yielding higher EQE than the cell with SiNx ARC in the short wavelength range (Figure 5h). Conversely, the solar cell's EQE values with double‐nanoblock LARC show superior values all over the spectra compared to all the others (Figure 5h). Figure 5i shows the EQE of the solar cell with double‐nanoblock LARC by changing the incident angle from 0 to 60° for s‐ and p‐ polarizations. Thanks to the broad angular responses of the Mie resonances, the cell does not exhibit appreciable dependence on the angle.

Si‐polymer hybrid heterojunction ultrathin Si solar cells, due to a low fabrication cost and high PCE attainable, have attracted large interests in recent years.^[^ ^19^, ^54^, ^55^, ^56^ ^]^ The n‐type c‐Si works as a light‐trapping and carrier generation layer in these structures. At the same time, the polymer acts as a hole transport layer. The scalable manufacturing of these cells is economical as it requires spin‐coating and direct annealing steps to form Si‐polymer heterojunctions. At the same time, the polymer also functionalizes as a passivation layer to reduce carrier recombination. Because of the limited light absorption into the active c‐Si thin film layer, designing efficient light‐trapping is essential to obtain efficient performances from these cells.

**Figure 6:**
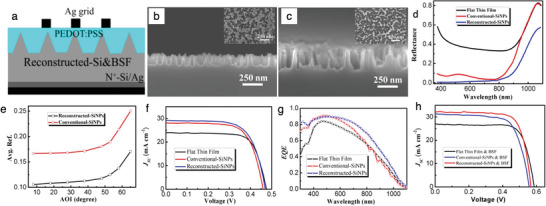
a) Device structure of thin film c‐Si/PEDOT:PSS hybrid solar cells with surface texturing of reconstructed‐SiNPs combined with highly doped BSF layer (N^+^‐Si). b) Cross‐sectional SEM images of SiNPs after the first round of the MaCE process and c) after the second round of the MaCE process. The insets in (b,c) show the top view of SiNPs after the first and second rounds of the MaCE process, respectively. d) Reflection spectra of 20 µm thick thin film c‐Si with flat, conventional‐SiNP, and reconstructed‐SiNP surface texturing in the whole wavelength range. e) Averaged reflectance in the full wavelength range of conventional‐SiNPs and reconstructed‐SiNPs at different AOIs. f) Current density–voltage (*J* − *V*) curves of thin film Si/PEDOT:PSS HHSCs with different surface texturing. g) EQE measurements results of thin film Si/PEDOT:PSS HHSCs with different surface texturing. h) *J* − *V* curves of the BSF‐combined 20 µm thick c‐Si/PEDOT:PSS HHSCs with and without surface texture. Reproduced with permission.^[^ ^57^ ^]^ Copyright 2015, American Chemical Society.

He et al. develop a hierarchical textured structure on a 20 µm c‐Si, combining with a hole transport layer of poly(3,4‐ethylene dioxythiophene): poly(styrenesulfonate) (PEDOT:PSS) to fabricate hybrid heterojunction solar cells (HHSCs).^[^ ^57^ ^]^ The cell manufacturing starts by reducing the Si wafer thickness from 270 to 20 µm by a concentration of 50 wt. % KOH solutions at 80 °C, followed by etching on the side walls to engineer a reconstructed surface as a light‐trapping layer. The rear side comprises a highly doped N^+^‐Si back surface field (BSF) layer. In this work, the authors compare textured cells against a flat thin film, a conventionally nanoporestructured (conventional‐SiNPs), and conventional‐SiNPs via a chemical reconstruction process (reconstructed‐SiNPs). **Figure** **6a** shows the schematic of the designed solar cell with reconstructed‐SiNPs. Figure 6b,c show the SEM images of SiNPs after the first and second rounds of the metal‐assisted chemical etching (MaCE) process. After the second round of etching, compared with the morphology observed in Figure 6b, the feature size of reconstructed SiNPs becomes larger. At the same time, their diameter decreases (Figure 6c).

Figure 6d shows the reflection spectra results of 20 µm thick film c‐Si of flat (black line), with textures of conventional‐SiNPs (red line), and with textures of reconstructed‐SiNPs (blue line). The conventional‐SiNPs sample shows a reflection lower than 10% in the 400–800 nm visible light wavelength region. At the same time, it reports an increased reflection beyond 800 nm wavelength, reaching similar values of the flat thin film (Figure 6d). Compared with the conventional SiNPs sample, the sample of reconstructed SiNPs shows a significant reflectance decrease over the whole wavelength (Figure 6d). Because of the second‐order nanostructures on the apex, there is a reduction of reflection in the region of near‐ultraviolet wavelength. In contrast, the enhancement of absorption in the NIR/infrared wavelength region arises from the effect of impedance‐matching, which originates from the bowl‐like structure in the texture. Figure 6e shows the averaged reflectance of conventional‐SiNPs (red line with circle hollow dots) and reconstructed‐SiNPs (black line with square hollow dots) at different incidence angles, showing the omnidirectional light‐harvesting ability of reconstructed‐SiNPs cells.

Figure 6f shows the *J* − *V* curve of thin film Si/PEDOT:PSS HHSCs against a flat thin film (black line), a conventional SiNPs (red line), and a reconstructed SiNPs cell (blue line). The system with reconstructed‐SiNPs shows a *J*_*SC*_ of 29.1 mA cm^−2^, *V*_*OC*_ of 469.2 mV, *FF* of 67.8%, and PCE of 9.27%. The cell with conventional‐SiNPs, conversely, reports a lower *J*_*SC*_ of 28.1 mA cm^−2^, indicating that reconstructed‐SiNPs provide stronger light‐trapping. Figure 6g shows the EQE of thin film Si/PEDOT:PSS HHSCs against a flat thin film (black line with square hollow dots), conventional‐SiNPs (red line with circle hollow dots) and reconstructed‐SiNPs (blue line with triangular hollow dots). The EQE of the Si/PEDOT:PSS HHSCs with reconstructed‐SiNPs shows superior EQE values over the entire wavelength range, resulting from the enhanced absorption in the NIR region with wavelength from 900 to 1100 nm and the wavelength below 400 nm in the short wavelength. In this work, the authors implement a c‐Si/PEDOT:PSS HHSCs cell combined with a BSF layer. Figure 6h reports the measurement result of the *J* − *V* curves about the c‐Si/PEDOT:PSS HHSCs. The reconstructed‐SiNPs textured 20 µm thick c‐Si/PEDOT:PSS HHSCs combined with BSF realize a PCE of 13.6%.

**Figure 7:**
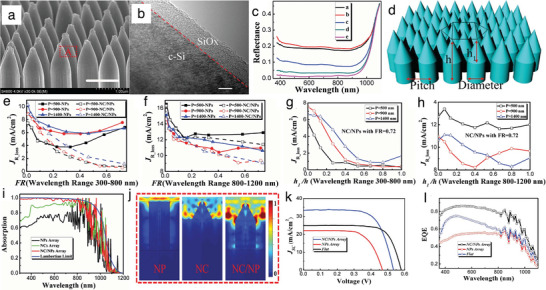
a) SEM images of the NC‐NPs array after the fourth‐round chemical reconstruction. b) The scale bar in (a) is 1 µm, and in (b) is 5 nm. c) Reflection curves of reconstructed NPs arrays with different etching cycles. d) Schematics of hexagonal arrays of Si nanostructures with NC‐NPs configurations and individual hexagonal structures. e,f) Optical photocurrent losses $J_{R\_loss}$ variation of NPs and NC‐NPs arrays with different FR and different P in the wavelength range of (e) 300–800 nm and (f) 800–1200 nm. g–i) $J_{R\_loss}$ variation of NC‐NPs arrays with different NCs heights in the wavelength range of (g) 300–800 nm and (h) 800–1200 nm. i) Simulated absorption curves of NPs, NCs, and NC‐NPs array with 10 µm‐thick c‐Si substrate and a Lambertian limit of Si film with equal thickness. j) NP, NC, and NC‐NP structure absorption profiles at the wavelength of 400 nm. k) Current–voltage *J* − *V* curves and l) EQE characteristics of the three types of solar cells. Reproduced with permission.^[^ ^20^ ^]^ Copyright 2016, Wiley‐VCH.

He et al. explores HHSCs engineered with NC‐NPs dual‐structured arrays on 20 µm c‐Si as a light‐trapping layer, combined with a PEDOT:PSS. The authors implement NC‐NPs arrays based on nanosphere colloidal lithography and MaCE techniques.^[^ ^20^ ^]^ **Figure** **7a** shows the SEM image of a fabricated NC‐NPs array. For each NC‐NP structure, the height of the NC at the top is 900 nm, while the height of the NP at the bottom is 600 nm. The top cone regions possess a polished surface, and the bottom pillar regions are perpendicular to the substrate. Figure 7b shows the surface morphology of a NC characterized by a high‐resolution transmission electron microscopy (HR‐TEM). The image shows that the surface remains atomically smooth with good‐quality Si crystalline structures.

Figure 7c illustrates the reflection characteristics of reconstructed NPs arrays fabricated by different MaCE cycles. The c‐Si with structures in Figure 7a shows an average reflectance of 1.5% in the wavelength range from 400  to 900 nm (Figure 7c), reporting one of the most robust anti‐reflection properties compared to other methods requiring ten times thicker nanostructures to achieve similar reflection values.^[^ ^51^, ^58^ ^]^

The authors perform simulations with finite‐elements solvers to investigate the mechanisms of light‐matter interactions between NC and NP. They study the relationship between the optical properties of surface texture and the photovoltaic performance by calculating the optical photo‐current losses ($J_{R\_loss}$) from surface reflectance^[^ ^59^ ^]^:

(3) $$J_{R\_loss}=\int_{\lambda_{min}}^{\lambda_{max}}\frac{e\lambda }{hc}R\left(\lambda \right)\times \phi_{AM1.5}\left(\lambda \right)\;d\lambda \;$$

where ϕ_*AM*1.5_(λ) is the standard sun power density. *R*(λ) is the reflectivity, *c* is the light speed in free space, *h* is the Plank constant, *e* is the unit charge, λ_*min*_, and λ_*max*_ are the minimum and the maximum illumination wavelengths. Figure 7d shows the schematic of NC‐NPs configurations. The authors perform simulations between 300 and 800 nm wavelengths and 800  and 1200 nm wavelengths. Figure 7e,f reports the $J_{R\_loss}$ calculations of NC‐NPs and NPs arrays with different pitch (P) and different filling ratios (FR). Structures with FR below 0.1 and P between 900 (red line with circle dots and red line with circle hollow dots in Figure 7e) and 1400 nm (blue line with triangular dots and blue line with triangular hollow dots in Figure 7e), together with structures possessing FR below 0.17 and P of 500 nm (black line with square dots and black line with square hollow dots in Figure 7e), show negligible $J_{R\_loss}$ differences in both the NC‐NPs and NPs arrays (Figure 7e), demonstrating their structural insensitivity at low FR.

From the results of Figure 7e, we observe that the $J_{R\_loss}$ of NC‐NP arrays decreases with increasing FR (lines with hollow dots). The $J_{R\_loss}$ of NPs arrays, conversely, first decreases with increasing the FR and then increases slightly with increasing the FR, saturating at a minimum value for different P (lines with dots in Figure 7e). These performances result from the fact that in NC‐NPs arrays, there is a graded effective refractive index transition between the air and the nanostructures, which yields superior anti‐reflection behavior. Between the wavelengths of 800  and 1200 nm, the NPs arrays show insensitive properties for light‐trapping when FR is higher than 0.1 (lines with dots in Figure 7f). Conversely, the NC‐NPs arrays offer enhanced light‐trapping when the FR increases (lines with hollow dots in Figure 7f). The authors attribute this result to the gradient variation of the effective refractive index between the bulk c‐Si and the top surface for the NC‐NPs arrays.

The authors compute the $J_{R\_loss}$ in NC‐NPs arrays. They vary the height of NC from 0  to 1500 nm and fix the FR = 0.72, computing their $J_{R\_loss}$ in the wavelength windows of 300 –800 nm and 800 –1200 nm. Figure 7g,h report the results. In the 300 –800 nm wavelength range, the $J_{R\_loss}$ decreases with increasing normalized NC height (r = *h*_1_/h) changing from 0 to 0.5 and saturates to a constant value for r up to 1.0. The NC‐NPs arrays with P of 500 nm (black line with square hollow dots in Figure 7g) and 900 nm (red line with circle hollow dots in Figure 7g), conversely, exhibit lower $J_{R\_loss}$ compared with P = 1400 nm (blue line with triangular hollow dots in Figure 7g). In the wavelength range of 800–1200 nm, when P = 500 nm, the NC‐NPs array has the largest $J_{R\_loss}$ (black line with square hollow dots in Figure 7h). These results imply that designs strongly impact light absorption when the pitches are comparable to the wavelength of incident light.

Figure 7i reports the simulation results of absorption curves for the Lambertian performance of Si film with equal thickness (blue line), NC‐NPs (red line), NCs (green line), and NPs array (black line) with c‐Si substrate of 10 µm‐thick. The c‐Si thin film textured with NC‐NPs array shows stronger light absorption than c‐Si thin film textured with NPs and NCs arrays, nearly approaching the Lambertian performance between the wavelengths from 300  to 800 nm.

Figure 7j shows the normalized electric field intensity (|*E*/*E*_0_|) distribution at the wavelength of 400 nm for NP (left), NC (middle), and NC‐NP (right) structure. For the c‐Si thin film textured with NPs array, the strong electrical field intensity distributed on the pillar top surface (Figure 7j). It implies that the location of the strong reflectance occurs at the pillar's top surface. In contrast, the c‐Si thin film textured with NC‐NPs and NCs array possess strong electrical field intensity distributed near the structure's tips and corners (Figure 7j). This result shows that the reflectance effectively suppresses in NC‐NPs array and NCs array driven by the morphology of the top surface varying from planar to tapered.

The authors further fabricated PEDOT:PSS/Si hybrid solar cells and compared them with a flat c‐Si surface, NPs array, and NC‐NPs array. Figure 7k shows the *J* − *V* curves of the PEDOT:PSS/Si HHSCs manufactured on 20 µm‐thick c‐Si substrates and with NPs arrays (red line) and NC‐NPs (blue line) textures, against flat c‐Si with 300 µm‐thick (black line) as reference. Measurement results show that the hybrid solar cell with NC‐NPs array achieves *V*_*OC*_, *J*_*SC*_, *FF*, and PCE of 0.531 V, 33.7 mA cm^−2^, 68.2%, and 12.2%, respectively. The PCE value from hybrid solar cells with NC‐NPs array shows an enhancement of 22% and 72% compared with the hybrid solar cells with flat surface and NPs array texture. The authors conclude that the best PV performance of hybrid solar cell with NC‐NPs array originate from the high *J*_*SC*_ based on superior light‐trapping property and moderate *V*_*OC*_, which is related to the dense PEDOT:PSS coverage on c‐Si and atomically smooth surface. Figure 7l shows the EQE results of the PEDOT:PSS/Si hybrid solar cell with a flat c‐Si surface (blue line with triangular hollow point), NPs array (red line with circle hollow point), and NC‐NPs array (black line with square hollow point). The PEDOT:PSS/Si hybrid solar cell with NC‐NPs arrays has better EQE than PEDOT:PSS/Si hybrid solar cell with NPs array over the entire wavelength range (Figure 7l).

## 3 Plasmonic Light Management

**Figure 8:**
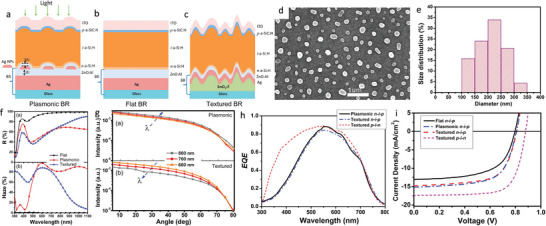
a–c) Schematic device structures of the n‐i‐p a‐Si:H solar cells deposited on the (a) plasmonic, (b) flat, and (c) textured BRs. SEM image (d) and size distribution (e) of Ag NPs. f) Total reflectance R and haze in reflection of flat, plasmonic, and textured BRs. g) Angular intensity distribution of light scattered by plasmonic and textured BRs at the wavelength of 600, 700, and 800 nm. h) EQE curves of plasmonic and textured n‐i‐p a‐Si: solar cells and textured p‐i‐n a‐Si:H solar cell fabricated on the Asahi VU‐type glass. i) *J* − *V* curves of flat, plasmonic, and textured n‐i‐p a‐Si:H solar cells, and textured p‐i‐n a‐Si:H solar cells. Reproduced with permission.^[^ ^60^ ^]^ Copyright 2012, American Chemical Society.

In addition to light‐trapping strategies with Si nanostructures, researchers investigate ultrathin Si solar cells with plasmonic textures.^[^ ^8^, ^47^, ^60^, ^61^, ^62^, ^63^, ^64^ ^]^ In general, to avoid the suppression of the photocurrent generated from the wavelength below surface plasmon resonance,^[^ ^65^, ^66^, ^67^, ^68^ ^]^ most of the previous works engineered plasmonic structures at the rear side of ultrathin solar cell. Tan et al. developed a plasmonic back reflector (BR) light‐trapping strategy by placing silver nanoparticles (Ag NPs)^[^ ^60^ ^]^ in n‐i‐p hydrogenated amorphous Si (a‐Si:H) solar cell. **Figure** **8a–c** shows the schematic structure of the solar cell with plasmonic BR, flat BR, and textured BR. The plasmonic BR manufacturing includes depositing 12 nm Ag film on the Ag/AZO layer after annealing at 400 °C for one hour in a vacuum oven. Figure 8d,e shows the SEM image and statistic of the Ag NPs size. These AgNPs possess an average equivalent diameter of 205 nm and a surface coverage of 16% (Figure 8d). Almost no small particles with a diameter<100 nm are observed (Figure 8e). The fabrication method improves previous manufacturing approaches,^[^ ^69^, ^70^ ^]^ which create smaller Ag NPs with a diameter lower than 100 nm and aggregates with relatively large surface coverage more extensive than 30%, yielding weak scattering and more parasitic absorption loss.

The plasmonic BR provides scattering and diffuse reflection to light not absorbed during a single pass into the solar cell. The angular intensity distribution (AID) and the haze, which is the ratio of diffuse to total reflection, are the two main parameters employed to characterize the light scattering properties of a back reflector.^[^ ^71^, ^72^ ^]^ Figure 8f shows the total reflectance R and haze in reflection of flat (black line with circle dots), plasmonic (red ine with square dots), and textured BRs (blue line with triangular dots). The haze parameter increases from zero value in a flat BR (Figure 8f) to a high value at short wavelengths in the textured BR, decreasing sharply with increasing wavelength above 600 nm. The plasmonic BR exhibits a haze above 80% in the wavelength range of 520–1100 nm (Figure 8f), opening applications either for standard a‐Si:H solar cells, which require light‐trapping wavelength up to 750 nm, or for lower bandgap absorbers. Figure 8g shows the AID of plasmonic and textured BRs at the wavelength of 600 (orange line with triangular dots), 700 (red line with circle dots), and 800 nm (grey line with square dots). The AID of textured BR and plasmonic BR show a similar trend, decreasing with an increase in the angle. Conversely, plasmonic BR shows superior scattered light intensity than textured BR at each wavelength (Figure 8g).

In contrast to textured BR, whose AID decreases with wavelengths ranging from 600 to 800 nm, the AID of plasmonic BR increases along with varying wavelengths from 600 to 800 nm (Figure 8g). This behavior originates from different scattering mechanisms of textured and plasmonic BRs. While a textured BR relies on surface roughness, a plasmonic BR provides stronger scattering from localized surface plasmon resonances of Ag NPs.

Figure 8h shows the EQE results of the p‐i‐n a‐Si:H solar cells with textures (red dashed line) n‐i‐p a‐Si:H solar cells with textures (blue dashed line) and n‐i‐p a‐Si:H solar cells with plasmonic BR (black line). The p‐i‐n a‐Si:H solar cells with textures show higher spectra efficiency at shorter wavelengths than the n‐i‐p a‐Si:H solar cells with textures (Figure 8h). The authors explain this result because of many factors: the absence of i/p interface optimizations compared to p‐i‐n configurations, higher absorption losses from the thicker p‐layer, and higher surface reflection of n‐i‐p solar cells. Figure 8i shows the *J* − *V* curve of flat (black line), n‐i‐p a‐Si:H solar cells with plasmonic BR (blue dashed line), and n‐i‐p a‐Si:H solar cells with textures (red dashed line) compared to a p‐i‐n a‐Si:H solar cells with textures (magenta dashed line). The work focused on measuring the performances of *J*_*sc*_ at wavelengths larger than 550 nm, because for λ<550 the back reflector scattering effect is inhibited by plasmonic absorption. The measurements show that the *J*_*sc* > 550*nm*_ of n‐i‐p a‐Si:H solar cells with plasmonic BR is 0.3 mA cm^−2^ lower than the textured p‐i‐n solar cell (Figure 8i). The PCE of the plasmonic BR‐based solar cell is 7.9%. A contribution to the lower performance outside the BR‐based absorption layer also comes from amorphous silicon, which has a higher bandgap than crystalline Si and sustains a lower current efficiency generation.

## 4 Conclusion

This review summarizes current progress and challenges in optical mechanisms for photon management in ultrathin Si‐based solar cells, including standard Si junctions and polymer‐based hybrid heterojunction devices. Light‐trapping structures in ultrathin Si solar cells aim to increase the optical path length through anti‐reflection coatings and light‐scattering layers. Most designs focus on manufacturing diverse implementations of anti‐reflection layers on the top surface of the device, incorporating Ag, Al, or SiO_2_ as back reflectors. Compared with conventionally Si solar cells with ARC of few micron thicknesses, the strategies developed for ultrathin solar cells demonstrated designs reaching equivalent visible light absorption in thickness as low as 450 nm.

However, present structured surfaces possess the shortcoming of a deep junction depth. A deeper junction sustains severe Auger and surface charge recombination losses, which hampers the final device performance. Pioneering work has proposed to address this issue by manufacturing solar cells within a few hundred‐nanometer thickness,^[^ ^5^ ^]^ with a total surface area 67% more extensive than a planar structure. Recent work also explored advanced texturing for light‐trapping, optimizing simultaneously anti‐reflection properties and light‐trapping for increased absorption into a single design structure. The increased light path from engineered scattering effects lowers the device thickness while maintaining sufficiently high performances.^[^ ^24^ ^]^ The present limitation of these approaches is their industrial scalability. These designs demand elaborate nanostructure patterns that currently require e‐beam nanolithography. Future directions in this research area are to scale these designs into cost‐effective light management textures with the same light absorption properties.

From the manufacturing point of view, functional heterojunction in ultrathin Si‐polymer hybrid solar cells are particularly attractive as they rely on simple fabrication steps comprising room temperature spin‐coating and short‐time low‐temperature annealing. However, the scalability requires the addition of development towards slot‐die coating or similar techniques. As to these ultrathin Si‐polymer hybrid solar cells, because Si acts simultaneously as a light absorber and carrier generator, it is critical to balance strong absorption effects with reduced surface area to decrease carrier recombination losses. Ultrathin Si‐polymer HHSCs can reach a comparable PCE of >10% of Si‐textured ultrathin solar modules.

Despite the various challenges in further optimizing photon harvesting in such small semiconductor volumes, research and development in ultrathin solar cells provide bright opportunities. As of 2019, the industry of Si consumes more than eight billion metric tons of semiconductor material for different applications. Predictions in the coming years forecast that this number will increase steadily and with a 6.5% rise by 2023.^[^ ^73^ ^]^ With large populations and rapid modernization, such a sharp change increase could strain the global demand‐supply balance for the Si manufacturing industry, with substantial repercussions on diverse markets, including energy, where Si is the most common semiconductor material used in 95% of commercial solar cells available. Photon management platforms that enhance the performances of ultrathin cells to a level comparable to commercially available bulk devices will save 1‐2 orders of magnitude amount of Si in each final module, providing a scalable roadmap to launch economic and performing renewable energy cells for different addressable markets in the next future.

## Conflict of Interest

The authors declare no conflict of interest.
